# HIV-1 Replication Is Differentially Regulated by Distinct Clinical Strains of *Mycobacterium tuberculosis*

**DOI:** 10.1371/journal.pone.0006116

**Published:** 2009-07-01

**Authors:** Shahin Ranjbar, Helena I. Boshoff, Amara Mulder, Noman Siddiqi, Eric J. Rubin, Anne E. Goldfeld

**Affiliations:** 1 Immune Disease Institute, Boston, Harvard Medical School, Boston, Massachusetts, United States of America; 2 The Tuberculosis Research Section, National Institute of Allergy and Infectious Diseases (NIAID), National Institutes of Health (NIH), Bethesda, Maryland, United States of America; 3 Department of Immunology and Infectious Diseases, Harvard School of Public Health, Boston, Massachusetts, United States of America; Karolinska Institutet, Sweden

## Abstract

**Background:**

Tuberculosis (TB) is the largest cause of death in human immunodeficiency virus type 1 (HIV-1) infection, having claimed an estimated one third to one half of the 30 million AIDS deaths that have occurred worldwide. Different strains of *Mycobacterium tuberculosis* (MTb), the causative agent of TB, are known to modify the host immune response in a strain-specific manner. However, a MTb strain-specific impact upon the regulation of HIV-1 replication has not previously been established.

**Methology/Principal Findings:**

We isolated normal human peripheral blood mononuclear cells (PBMC) and co-infected them with HIV-1 and with either the well characterized CDC1551 or HN878 MTb clinical isolate. We show that HIV-1 co-infection with the CDC1551 MTb strain results in higher levels of virus replication relative to co-infection with the HN878 MTb strain *ex vivo*. Furthermore, we show that the distinct pattern of CDC1551 or HN878 induced HIV-1 replication is associated with significantly increased levels of TNF and IL-6, and of the transcription and nuclear translocation of the p65 subunit of the transcription factor NF-κB, by CDC1551 relative to HN878.

**Conclusions/Significance:**

These results provide a precedent for TB strain-specific effects upon HIV-1 replication and thus for TB strain-specific pathogenesis in the outcome of HIV-1/TB co-infection. MTb strain-specific factors and mechanisms involved in the regulation of HIV-1 during co-infection will be of importance in understanding the basic pathogenesis of HIV-1/TB co-infection.

## Introduction

*Mycobacterium tuberculosis* (MTb), the causative agent of tuberculosis (TB) disease, is the most frequent co-infection in human immune deficiency virus type 1 (HIV-1) infected patients. Furthermore, TB is thought to have caused a third to a half of all acquired immune deficiency syndrome (AIDS) deaths that have occurred to date [1], [2], particularly in sub-Saharan Africa and South East Asia [2], [3], areas of world where HIV-1 infection is expanding most rapidly.

TB in the setting of HIV-1 infection causes increased mortality in part due to its activation of the innate and adaptive immune system and subsequent production of specific cytokines and intracellular signal transduction pathways resulting in the induction of HIV-1 replication [4], [5], [6], [7]. Increased HIV-1 replication in turn leads to higher viral loads, CD4+ T cell destruction, and higher mortality in co-infected patients [7], [8]. Moreover, the progressive immune compromise associated with HIV-1 infection and AIDS results in reactivation of TB disease in latently infected individuals, and an increase in primary TB and secondary TB infection [8], [9], [10], [11]. Notably, in severely immune compromised AIDS patients, there is an extremely high mortality within the first months after a diagnosis of TB [8], [12], [13], particularly if the TB is drug resistant [14].

There is significant variation in MTb clinical isolates [15], [16], [17]. The clinical strains CDC1551 and HN878 provide two well-characterized examples of clinical MTb isolates that have a distinct impact upon the host immune response. CDC1551 was originally characterized as hypervirulent due to its extensive transmission from close and casual contacts [18]. However, when compared to another clinical MTb isolate, HN878, in infected mouse lung and in bone marrow derived macrophages (BMM), the CDC1551 strain elicited a more vigorous pro-inflammatory cytokine response [19], [20]. The HN878 strain is significantly more lethal than CDC1551 in murine TB models, and this lethality has been attributed to a relative failure of HN878 to induce a prompt and effective Th1-type immune response as compared to CDC1551 [19], [20], [21]. The hypervirulent phenotype of HN878 has also been associated with specific differences in lipid components of its cell wall (reviewed in: [22]), specifically to the production and secretion of a highly active lipid-species-a polyketide synthase (*pks15/1*)-derived phenolic glycolipid (PGL), which dampened the host's proinflammatory cytokine response during TB infection in a mouse model [20].

While MTb strain variation appears to affect the course of TB, does it also differentially regulate HIV-1 replication? Using an *ex vivo* HIV-1/TB co-infection model, here we show that infection of human peripheral blood mononuclear cells (PBMC) with the CDC1551 MTb strain causes significantly higher viral replication of clinical isolates representative of HIV-1 subtypes B, C, and E, as compared to infection with HN878. Furthermore, we show that deletion of PGL in the mutant HN878 strain results in a minimal up-regulation of HIV-1 replication. Finally, we demonstrate that the mechanism for this differential regulation of HIV-1 involves differential production of TNF and IL-6 and the transcription and nuclear localization of p65, the activating subunit of the NF-κB complex. These results provide evidence that HIV-1 regulation is influenced by TB strain type in HIV-1/TB co-infection and may thus provide insight into specific patterns of lethality in certain TB outbreaks in patients with AIDS.

## Results

### CDC1551 infection results in higher HIV-1 replication as compared to infection with HN878

We infected freshly isolated PBMC from eight normal PPD positive donors with the MTb strains CDC1551 or HN878 overnight and infected the cultures with dual tropic (X4R5) viruses that use both CXCR4 and CCR5 co- receptors for cellular entry, from subtype B, C, and E HIV-1 (HIV-1_89.6_, B subtype; HIV-1_98IN17_, C subtype; and HIV-1_93TH51_, E subtype). Free virus levels in the cellular supernatants were assessed at day 3, 7, and 10 post-HIV-1-infection. In order to maximize the duration of cell survival under co-infection conditions and thus observe maximal differences in viral replication, we infected the cultures with a low concentration of HIV-1 (50 TCID_50_).

**Figure 1:**
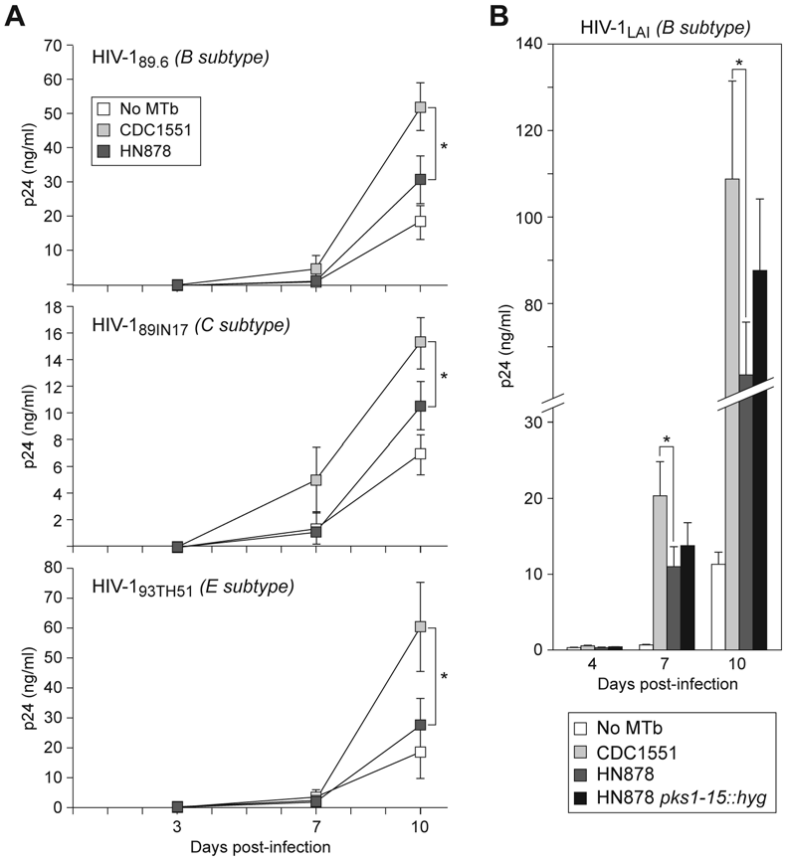
HIV-1 replication is differentially regulated by different strains of MTb. (A) HIV-1 p24 levels produced by co-infection of PBMC with different TB strains and X4R5 viruses from subtype B (HIV-1_89.6_), C (HIV-1_98IN17_) and E (HIV-1_92TH51_). PBMC from eight different donors were infected in duplicate with the MTb strain CDC1551 or HN878 and then infected with HIV-1_89.6_, HIV-1_98IN17_, and HIV-1_92TH51,_ the next day. Virus replication was significantly higher in the cultures co-infected with the MTb strain CDC1551 as compared to HN878 at day 10 post-infection with HIV-1_89.6_, HIV-1_98IN17_ and HIV-1_92TH51_ (*, p<0.05). Results are shown as mean±SEM. (B). HIV-1 p24 levels produced by co-infection of PBMC with different TB strains and X4 HIV-1_LAI_ (B subtype). PBMC from eight different donors were infected in duplicate with the MTb strain CDC1551, HN878, or HN878 *pks1-15::hyg* and then infected with HIV-1_LAI_. Virus replication was significantly higher in the cultures co-infected with the MTb strain CDC1551 as compared to HN878 (*, p≤0.05 at day 7 and 10 post-infection). In the cultures co-infected with HN878 *pks1-15::hyg*, virus replication was non-significantly higher at day 7 (1.3 fold) and day 10 (1.4 fold) as compared to the cultures co-infected with wild type HN878. Results are shown as mean±SEM.

HIV-1 replication was higher in all cultures co-infected with MTb (all three strains) compared to cultures infected with HIV-1 alone (Figure 1A). Strikingly, CDC1551 infection of PBMC resulted in significantly higher levels of HIV-1 replication at day 10 post-HIV-1-infection of all three HIV-1 subtypes (p≤0.05) (Figure 1A). Thus, different MTb strains distinctly regulate the dual tropic subtype B, C, and E HIV-1 isolates tested. We next investigated whether a CXCR4 dependent T cell tropic HIV-1 isolate (HIV-1_LAI_) was also differentially regulated by co-infection with CDC1551 and HN878. We found that, p24 levels were significantly higher in CDC1551 infected cultures using HIV-1_LAI_ (Figure 1B) similar to our results using dual tropic X4R5 viruses.

Since previous studies showed that the PGL from HN878 inhibited inflammatory cytokine production in murine bone marrow macrophages (BMM) [20], we next tested whether a mutant strain of HN878 (HN878 *pks1-15::hyg*) containing a deletion in the gene encoding this PGL differentially regulated HIV-1 as compared to the parental HN878 strain. In cultures co-infected with HN878 *pks1-15::hyg*, HIV-1 replication was non-significantly higher than in the cells co-infected with wild type HN878 (Figure 1B) suggesting that this distinct lipid in the HN878 cell wall is not a major determinant of differential HIV-1 replication in this system. We analyzed the formation of colony-forming units (CFU) after infection by both bacterial strains and found no significant difference in bacterial number at day 4 and 7 post infection of the PBMC cultures (data not shown). We note that it has previously been shown that the *in vitro* growth rates of CDC1551 and HN878 are very similar [23]. Taken together, these results show that HIV-1 replication is differentially influenced by different MTb isolates.

### CDC1551 and HN878 differentially regulate the host immune response in human PBMC

Previous studies using murine lung and BMM showed that CDC1551 and HN878 differentially induced a number of host factors involved in the innate immune response including TNF, IL-10, IL-6, and IL-12 [19], [20], [21]. In human macrophages it was also shown that TNF and IL-12 were differentially induced by CDC1551 and HN878 [24]. Furthermore, in murine cells it was shown that the HN878 PGL was critical in the regulation of TNF, IL-6, and MCP-1 since deletion of the PGL in the HN878 mutant (HN878 *pks1-15::hyg*) resulted in higher levels of these factors, which was associated with enhanced survival of TB infected mice [20].

**Figure 2:**
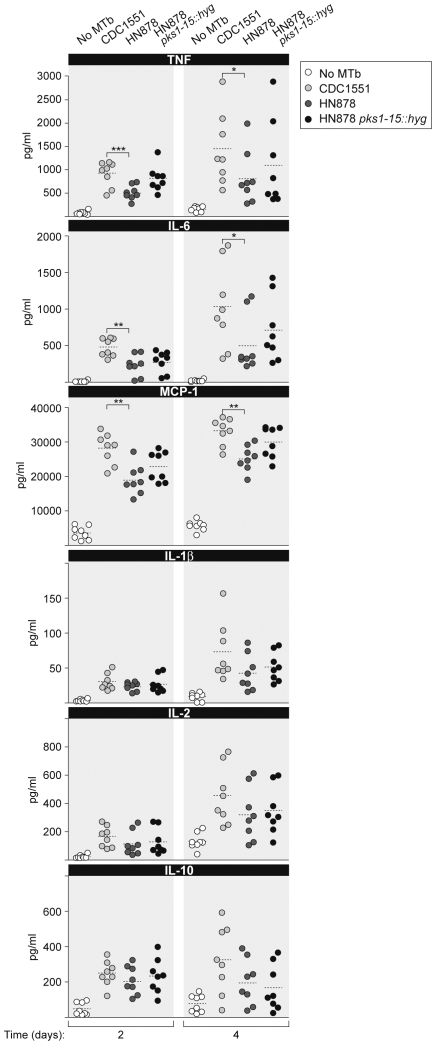
CDC1551 and HN878 infection differentially regulate TNF, IL-6 and MCP-1 in human PBMC. PBMC from eight different donors were infected in duplicate with the MTb strain CDC1551, HN878, or HN878 *pks1-15::hyg* for 2 or 4 days and levels of TNF, IL-6 and MCP-1 were assessed. Production of TNF, IL-6 and MCP-1 was significantly higher in CDC1551 infected cells as compared to HN878 infected cells (*p<0.05; **, p<0.01; ***, p<0.005). Infection of PBMC with HN878 *pks1-15::hyg* non-significantly increased the production of these cellular factors as compared to infection with HN878. Expression of IL-1β, IL-2 and IL-10 were also on average minimally higher in CDC1551 versus HN878 infected cells, but not significantly.

We thus next examined the levels of TNF, IL-1β, IL-2, IL-6, IL-10, and MCP-1 (also known as CCL2) in human PBMC infected by CDC1551, HN878, and HN878 *pks1-15::hyg* 2 and 4 days post-MTb infection. As shown in Figure 2, a significantly higher amount of TNF, IL-6, and MCP-1 was detected in supernatants from PBMC infected with CDC1551 as compared to cultures infected with HN878. Furthermore, infection with HN878 *pks1-15::hyg* resulted in non-significant increase of these cytokines as compared to wild type HN878 in each case (Figure 2). By contrast, we did not detect significantly higher levels of IL-1β, IL-2, and IL-10 in PBMC cultures infected with CDC1551 as compared to those infected with HN878 (Figure 2). To test whether co-infection with HIV-1 might influence cytokine levels in this co-infection model, we also evaluated cytokine levels in PBMC cultures co-infected with the three isolates of HIV-1 representing B, C, and E subtypes and with CDC1551 and HN878. We found no difference in the levels of cytokines measured (data not shown). Thus, the infecting TB strain is the determinant factor in the levels of TNF, IL-6 and MCP-1. CDC1551 infection results in significantly higher levels of these factors relative to HN878 infection. Similar to the lack of a significant effect upon HIV-1 replication, the PGL deletion in HN878 did not have a significant effect on the regulation of TNF, IL-6 and MCP-1 levels by HN878.

### Inhibition of CDC1551-induced TNF and IL-6 significantly decreases HIV-1 replication in a dose-dependent manner

**Figure 3:**
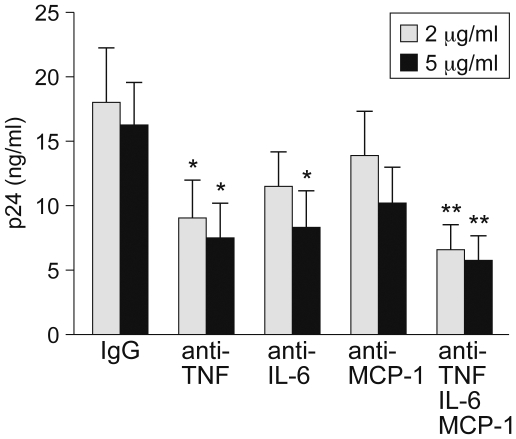
Blocking TNF or IL-6 decreases HIV-1 replication significantly in a dose dependent manner. Virus replication was determined by measuring HIV-1 p24 levels in the culture supernatants in PBMC from six individual donors at day 7 post-infection performed in duplicate. Blocking TNF (at 2 ug/ml or 5 ug/ml) or IL-6 (5 ug/ml) significantly decreased virus replication (*, p<0.05) as compared to the same concentrations of an IgG_1_ control antibody. Blocking of MCP-1 alone on average minimally decreased HIV-1 replication, but this did not reach statistical significance. Virus replication was further significantly inhibited in cultures containing a mixture of blocking antibodies to TNF, IL-6, and MCP-1 at a concentration of 2 µg/ml and 5 µg/ml (**, p<0.01). Results are shown as mean±SEM.

Since we observed differential induction of TNF, IL-6, and MCP-1 in human PBMC, all factors that increase HIV-1 replication [25], [26], [27], we next investigated the effect of blocking TNF, IL-6, or MCP-1 upon HIV-1 replication in CDC1551 co-infected cultures. Using PBMC from six donors we blocked TNF, IL-6, and MCP-1 function by increasing concentrations of neutralizing monoclonal antibodies to these proteins and used a mouse IgG1 isotype antibody as a control. The cells were then infected with CDC1551 and 42 hours later co-infected with HIV-1_LAI_. As shown in Figure 3, virus replication was significantly inhibited in a concentration-dependent manner by antibodies to TNF at 2 µg/ml (p<0.05) and IL-6 at 5 µg/ml (p<0.05) comparison to the non-specific effects of an isotype control antibody (Figure 3). Virus replication was further significantly decreased when a mixture of blocking antibodies to TNF, IL-6, and MCP-1 (at 2 ug/ml and 5 ug/ml of each antibody) was added to the cell cultures co-infected with CDC1551 and HIV-1 (p<0.01) (Figure 3). Blocking of MCP-1 lowered p24 levels but not significantly in comparison to the non-specific effects of an isotype control antibody (Figure 3). Thus, we conclude that in CDC1551-infected PBMC, the higher levels of TNF and IL-6 functionally contribute to the higher levels of p24 relative to PBMC infected with HN878.

### CDC1551, HN878 and HN878 *pks1-15::hyg* differentially regulate p65 transcription and nuclear translocation

One of the mechanisms by which MTb increases HIV-1 replication is by promoting the nuclear translocation of transcription factors required for activation of HIV-1 transcription via its long terminal repeat (LTR) [5], [28], [29]. Upon MTb ligation of Toll like receptors (TLR), several signal transduction cascades are stimulated, including the cascade leading to NF-κB translocation to the nucleus [30]. Furthermore, TNF and MCP-1 both increase nuclear NF−κB levels after engaging their own cognate receptors [30], [31], [32]. Since NF-κB is recruited to the HIV-1 LTR of all subtypes [5], [25], [33], [34], [35] and is a critical factor in HIV-1 replication, we thus next investigated the impact of MTb strain variation upon the transcription and nuclear translocation of the activating NF-κB subunit, p65.

**Figure 4:**
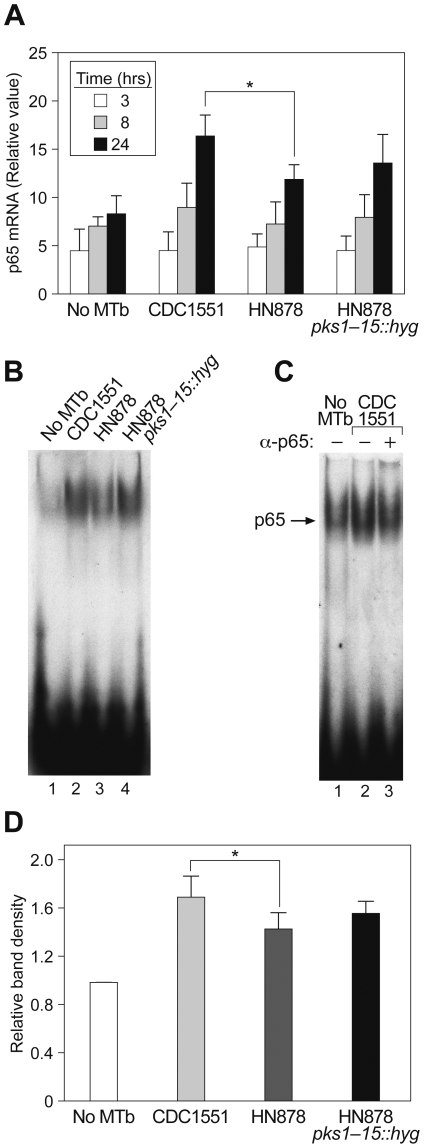
CDC1551 and HN878 differentially induce transcription and nuclear localization of the p65 subunit of NF-κB. (A) p65 mRNA levels in PBMC from six individual donors infected with CDC1551, HN878, or HN878 *pks1-15::hyg* for 3, 8 and 24 hours. There was no significant difference in p65 mRNA levels across uninfected cells and the cells infected with CDC1551, HN878, or HN878 *pks1-15::hyg* after 3 and 8 hours infection. A significant difference in p65 mRNA levels was observed 24 hours after infection in CDC1551 infected cells as compared to HN878 infected cells (*, p<0.05). (B) A representative EMSA analysis using nuclear extracts from PBMC infected with CDC1551, HN878, and HN878 *pks1-15::hyg* for 24 hours. DNA binding activity of NF-κB was higher in nuclear extracts prepared from cells infected with CDC1551 as compared to HN878 (lanes 2 and 3). Infection with HN878 *pks1-15::hyg* increased NF-κB DNA binding activity as compared to HN878. (lanes 2, 3 and 4). The gel shown is representative of EMSAs performed using nuclear extracts prepared from PBMC from six individual donors. (C) The induced complex seen on EMSA contains the activating p65 subunit of NF-κB. An antibody to p65 specifically reacted with the complex that is differentially increased by CDC1551 and HN878, specifically inhibiting the formation of the p65 complex in lane 3 relative to the complex in lane 2. When quantified by densitometric analysis the inhibition of the complex after antibody reaction was 57%. (D) Increased nuclear translocation of the p65 NF-κB subunit after infection with CDC1551 as compared to HN878. The histogram shows an average of the densitometric analyses of the p65 band from autoradiographs of the EMSAs from nuclear extracts prepared from the six donors whose PBMC were either uninfected or infected with CDC1551, HN878, or HN878 *pks1-15::hyg* as indicated. The results are shown as mean±SEM and demonstrate that there was significantly higher p65 levels in CDC1551 than in HN878 infected cells. (*, p<0.05).

We infected PBMC from six normal PPD-positive individuals with CDC1551, HN878 or HN878 *pks1-15::hyg* for 3, 8 and 24 hours and then extracted whole cell RNA and measured p65 mRNA levels using real time PCR. As shown in Figure 4A, there was not a significantly difference in p65 mRNA levels across the cells infected with the three MTb strains and uninfected cells at 3 and 8 hours post infection. However, CDC1551 infection significantly increased p65 mRNA levels compared to HN878 24 hours after infection (p<0.05). In addition, p65 mRNA levels were non-significantly higher in cells infected with HN878 *pks1-15::hyg* compared with cells infected with HN878 (Figure 4A).

We also investigated the nuclear translocation of p65 in PBMC from six PPD-positive normal donors infected by the three MTb strains for 24 hours. Using nuclear extracts prepared from individual donor PBMC, we performed EMSAs with an oligonucleotide matching the proximal NF-κB consensus motif from the HIV-1_LAI_ LTR. Figures 4B and 4C show a representative experiment from a single individual from six individuals' PBMC that were evaluated in this manner. We detected an inducible complex in cells infected with CDC1551 (Figure 4B, lane 2), which was demonstrated to be the p65 subunit by its reactivity with an antibody to p65 (Figure 4C, compare lanes 2 and 3). Furthermore, we detected an increase of p65 binding after infection with CDC1551 relative to HN878 (Figure 4B, compare lanes 2 and 3). We note that after infection with the HN878 mutant (HN878 *pks1-15::hyg)* there was increased levels of p65 compared to HN878, (Figure 4B, compare lanes 3 and 4). To quantify the increased binding in infected cells from the six individuals evaluated, we performed densitometric analysis of the p65 band from the autoradiographs of the EMSA experiments using the PBMC from the six individual donors (Figure 4D). This demonstrated that there were significantly higher p65 levels in CDC1551 infected cells compared to HN878 infected cells, and that the deletion of PGL from HN878 non-significantly increased p65 levels (Figure 4D), consistent with the experiments presented in Figures 4B and 4C.

## Discussion

Although MTb clinical strains with distinct immune modulatory characteristics have been identified, the effect of MTb phenotypic variation on HIV-1 replication and AIDS progression in co-infection is unknown. In this study we have shown for the first time that distinct activation of the host immune response by phenotypically distinct MTb clinical strains directly regulates HIV-1 replication in an *ex vivo* co-infection model.

Recognition of MTb by phagocytic cells leads to immune activation and induction of a complex series of interactions between various cell populations [36], [37], [38], which results in the production of a large number of cytokines and chemokines that are involved in TB and AIDS disease outcome in co-infected patients [4], [5], [39], [40], [41], [42]. Specifically, the induction of inflammatory molecules such as TNF, IL-6, and MCP-1 by active MTb infection is important for inhibition of TB disease progression [5], [43], [44], while at the same time these same molecules enhance HIV-1 replication [25], [26], [27], [34], [45], [46], [47] (Figure 3).

The binding of TNF as well as MCP-1 to their cognate receptors induces the activation of the IκB kinase complex and the nuclear translocation of active NF-κB [30], [31], [32], [48]. In addition, IL-6, TNF, and MCP-1 signaling triggers a cascade of events in the cell that results in the activation of the AP-1 transcription factor complex ([49], [50], [51]. In the nucleus, NF-κB and AP-1 bind to their cognate sites on the HIV-1 LTR and thereby enhance virus transcription [25], [33], [50], [51], [52], [53]. Furthermore, NF-κB and AP-1 serve as critical regulators of the inducible expression of many host factors that are important for upregulation of HIV-1 replication ([32], [49], [54], [55], and reviewed in [56]). Here, we have demonstrated that levels of TNF and IL-6 and the transcription and DNA binding of NF-κB is significantly and differentially regulated by infection by two distinct MTb strains, HN878 or CDC1551. These results demonstrate a correlation between infection by two distinct MTb strains and their differential effect upon the innate and adaptive immune system, which is consistent with their differential impact upon HIV-1 replication.

Transcription mediated by the HIV LTR depends on cooperative interactions among transcription factors and other cofactors, such as P-TEFb and Tat. Although the differential effect of CDC1551 and HN878 on virus replication is clearly multifactorial, given the importance of NF-κB in HIV-1 gene transcription, even a small effect upon its concentration and binding to the LTR could have a large functional impact [57], [58]. Thus, the demonstration that there is a significant difference between p65 mRNA and nuclear protein levels in CDC1551 and HN878 infected cells demonstrates one specific mechanism that might underlie differential activation of replication by the two TB strains.

It is thus reasonable to surmise that infection with phenotypically distinct MTb strains can potentially result in different viral loads *in vivo*, and to different patterns of disease progression in co-infection. Notably, intrapatient HIV-1 heterogeneity and diversity of virus quasispecies have previously been associated with TB/HIV-1 co-infection [39], [40]. An implication of the findings presented here may thus be that certain MTb strains such as CDC1551, which cause higher levels of HIV-1 replication relative to other MTb strains like HN878, may differentially enhance the emergence of relatively more virulent and drug-resistant HIV-1 isolates.

One of the main factors that determine the unique phenotypic characteristics of HN878 and CDC1551 strains is their distinct cell wall composition [20]. In animal models it has been shown that PGL, which is present in HN878 and not in CDC1551, plays an important role in hypoimmunogenicity and thus the hyperlethality associated with HN878 infection [20]. Here, we have shown that PGL is not a significant factor in virus replication elicited by HN878, although co-infection with HN878 *pks1-15::hyg* (lacking PGL production) was higher than that observed for HN878 and resulted in higher levels of p65 mRNA and nuclear localization of the factor itself. Thus, although it may play a role, our observations are consistent with the conclusion that the lower level of HIV-1 replication and cytokine and chemokine production elicited by HN878 as compared to CDC1551 is multifactorial and involves the differential expression of other MTb genes.

CDC1551 is a member of the Latino-American and Mediterranean (LAM) mycobacterial family and is a member of principal genetic group 2 (PGG 2) [59], [60]. By contrast, HN878 is a member of W-Beijing family of isolates [61], [62] and belongs to PGG 1 [63]. In 2006 a study from KwaZulu Natal (KZN), South Africa described an outbreak of extensively drug resistant (XDR)-TB in the context of advanced AIDS infection where 52 of 53 patients died very rapidly within a median time of 16 days post-TB diagnosis [14]. Subsequent genetic analysis of the bacterial strains causing this outbreak revealed that it was caused by another LAM family member the F15/LAM4/KZN strain [64], which also belongs to the principal genetic group 2 (PGG 2) [63]. Thus, it is intriguing to speculate that there are pathogenesis factors that are separate from drug resistance profiles that contribute to the high mortality in certain HIV-1/TB co-infected patients. Thus, we expect that future studies may identify additional mechanisms of direct interaction of HIV-1 with specific MTb clinical strains that will lead to understanding distinct patterns of pathogenesis in HIV-1/TB co-infected patients.

In summary, we have shown for the first time that infection by different MTb strains results in distinct levels of HIV-1 replication. We have linked this observation to the distinct regulation of cytokine expression, and to the activation of NF-κB. Thus, HIV-1 replication and progression to AIDS as well as TB disease outcome in HIV-1/TB co-infection is likely to reflect not only variable host factors, but also the variation of the MTb strains and expression of bacterial factors that interact with the host. Further investigation to determine MTb strain-specific factors and mechanisms responsible for the increase in AIDS and TB disease progression will be of importance in understanding basic mechanisms of HIV-1/TB pathogenesis.

## Materials and Methods

### MTb culture

MTb clinical strains CDC1551, HN878, and HN878 *pks1-15::hyg* were prepared by adding 50 µl of frozen bacteria stock into 10 ml of Middlebrook 7H9 medium (Difco BD, Franklin Lakes, NJ) supplemented with albumin dextrose complex (ADC) and 0.05% Tween 80 (Sigma-Aldrich, Saint Louis, MO). The growth rates of these MTb strains in *in vitro* culture is very similar, with HN878 showing a doubling time of 16.7 h and CDC1551 a doubling time of 17.1 h. [23]. Each strain was grown to an OD_650_ of 0.4 at 37°C, which assured that they were in the logarithmic growth phase. Cells were then pelleted and washed with PBS and re-suspended in RPMI 1640 and passed through a 5 µm filter to ensure that the bacteria were in a single cell suspension. Bacterial cell numbers were determined by measurement of OD_650_ before further dilution for cell infection studies and were plated 1.5×10^5^ bacteria/1.5×10^6^ cells.

### HIV-1 culture

HIV-1_LAI_ was obtained from The Centralized Facility for AIDS Reagents, National Institute for Biological Standard and Control (NIBSC), United Kingdom. HIV-1_89.6_, HIV-1_98IN17_, and HIV-1_93TH51_ were obtained from the NIH AIDS Research and Reference Reagent Program, USA. High titers of viral stocks were prepared by infection of a pool of normal human donor PBMCs stimulated with phytohemoglutinin (PHA) (Sigma-Aldrich) and IL-2 (AIDS Research and Reference Reagent Program). Supernatants containing high titers of viable virus were aliquoted and stored at −70°C.

### MTb and HIV-1 infection of cultured PBMC

PBMC were isolated from eight normal PPD positive donors and cultured in 24-well plates at 1.5×10^6^ in 1.5 ml RPMI 1640 medium (BioWhittaker, Inc., Walkersville, MD) supplemented with 10% heat-inactivated fetal calf serum (FCS) (Gemini Bio-Products, Woodland, CA) and 2 mM L-glutamine. Cells were immediately infected with 1.5×10^5^ MTb at a 1∶10 ratio of bacteria to cells and incubated at 37°C and 5% CO_2_ for approximately 10 hours. We stained the cells with CD14-FITC and performed flow cytometry analysis and found an average of 10% monocytes in samples from the donors used. The cultures were then mock infected or infected with 50 TCID_50_ HIV-1. Uninfected cultures and cultures infected with HIV-1 alone were also set up as controls. At day 2, 4, 7, and 10 post-infection, culture supernatants were collected and stored at −70°C until analysis.

### ELISA

Cytokine (IL-1β, IL-2, IL-6, IL-10, TNF) or chemokine (monocyte chemotactic protein-1 [MCP-1]) levels in cell culture supernatants were measured using OptEIA ELISA kits (BD-Pharmingen, Franklin Lakes, NJ). Cell-free virus levels were determined by measuring HIV-1 p24 antigen levels in culture supernatants using the HIV-1 p24 ELISA Kit (PerkinElmer, Boston, MA).

### Antibody neutralization assay

Increasing concentrations of neutralizing monoclonal antibodies to TNF, IL-6 (R&D Systems, Minneapolis, MN), MCP-1 (eBioscience, San Diego, CA), or mouse IgG isotype control (R&D Systems) were added to 1.5×10^6^ freshly isolated PBMC from six PPD-positive donors in complete RPMI 1640 media supplemented as above in 24-well plates just prior to infecting the cells with the MTb strain CDC1551 as described above. Antibody concentrations were first titrated to determine the maximum concentration that had no effect on cell viability and activation. Cultures were incubated at 37°C for 42 hours and then infected with HIV-1_LAI_ and cell-free virus levels were measured at day 7 post-HIV-1 infection as described above.

### Quantitative PCR

The mRNA expression level of the p65 subunit of NF-κB was determined by real-time PCR using SYBR green (Applied Biosystems, Foster City, CA). The reaction conditions were 95°C for 10 min followed by 40 cycles of 95°C for 20 sec and 60.5°C for 1 min. The results were normalized using β-actin mRNA as an internal control and expressed as relative values.

### Nuclear protein extract preparation and electrophoretic mobility shift assay

PBMC from six normal donors were infected with CDC1551, HN878, or H878 *pks1-15::hyg*, or mock infected as described above (1×10^7^cells per infection condition). After 24 hours, the cells were harvested, and nuclear extracts were prepared from infected and uninfected cells and passed through 0.2 µm filters to remove infectious MTb from the lysates. Electrophoretic mobility shift assays (EMSAs) were then performed as previously described [65], [66] using synthetic oligonucleotides corresponding to one of the NF-κB binding motifs in the HIV-1_LAI_ LTR (5′-CGCTGGGGACTTTCCAGG-3′). The anti-p65 antibody used in the supershift assays was purchased from Santa Cruz Biotechnology (SantaCruz, CA). Images of gels were captured using a Molecular Dynamics phosphorimaging system. Densitometric analysis was performed on the p65 complex using Quantity1 analysis (Biorad, CA) software. All values in each experiment were normalized to the p65 band in experiments with unstimulated cells. Results of the 6 individuals were averaged and are displayed with SEM.

### Statistical analysis

Where applicable, results are expressed as a mean±SEM. Comparison between two groups was performed using the paired Student t-Test with the aid of Microsoft Excel software. p≤0.05 was considered significant.
